# Supplementary material for: Nutrition and Diet Apps: Brazilian Panorama before and during the COVID-19 Pandemic
Source: Nutrients. 2023 Aug 17;15(16):3606. doi: 10.3390/nu15163606 (PMC10459095; doi:10.3390/nu15163606)
Supplement: Supplementary file 1 [file nutrients-15-03606-s001.zip › nutrients-2531372-supplementary.pdf]

Table S1 - Relative and absolute frequencies of data for all nutrition-related apps found on official Apple and Google Stores websites and platforms.

Variables				All (n=757)
Number of languages available by app				
01 – 10				96.6% (n=731)
11 – 20				2.5% (n=19)
21 – 30				0.5% (n=4)
> 30				0.4% (n=3)
Target population				
<18 years				95.6% (n=724)
>18 years				4.2% (n=32)
No information				0.1% (n=1)
Investment for use				
Free				17.8% (n=135)
Payment				59.0% (n=447)
No information				23.1% (n=175)
Prices				
Up to US$11.99				26.9% (n=204)
US$12 - $23,99				5.7% (n=43)
>US$24				6.1% (n=46)
No information				43.5% (n=329)
Seller				
One app				90.4% (n=587)
Two apps				6.8% (n=44)
Three apps				1.5% (n=10)
Four apps				1.3% (n=8)
Number of reviews	iOS device	Apple (Website)	Android device	Play Store (Website)
< 9.999	35.5%(n=269)	36.3% (n=275)	19% (n=144)	32.5(n=246)
10.000 – 19.999	1.7% (n=13)	1.1% (n=8)	2.4% (n=18)	2.1% (n=16)
> 20.000	5.4% (n=41)	4.8% (n=3)	6.5% (n=49)	8.9% (n=67)
No information	57.3% (n=434)	57.9%(n=438)	72.1% (n=546)	56.5% (n=428)
Number of downloads				
Up to 9.999				24.2% (n=183)
10.000 – 19.999				12.5% (n=95)
> 20.000				41.6% (n=315)
No information				21.7% (n=164)
Consumer rating	iOS device	Apple (Website)	Android device	Play Store (Website)
≤3.0	9.8% (n=74)	1.6% (n=12)	3% (n=23)	2% (n=15)

3.1 – 3.9	2.9% (n=22)	0.4% (n=3)	3.6% (n=27)	5.3% (n=40)
>4.0	47% (n=356)	42.3% (n=320)	21% (n=159)	29.5% (n=223)
No information	40.3% (n=305)	55.7% (n=422)	72.4% (n=548)	63.3% (n=479)
Technical support				
Yes				95.4% (n=722)
No				4% (n=30)
No information				0,7% (n=5)
Category				
Health and Fitness				80.1% (n=606)
Food and drink				11.5% (n=87)
Others [e]				7.7% (n=58)
No information				0.8% (n=6)
Release year				
Up to 2019				37.0% (n=280)
After 2019				54.3% (n=411)
No information				8.7% (n=66)

Obs. For sample definition (n), we considered only the app that gave this information.

Table S2 - Relative and absolute frequencies of data from nutrition-related applications that inform the year of launch on official Apple and Google Stores websites and platforms.

**Variables**	**Before 2019 (n=280)**	**After 2019 (n=411)**
Number of languages available by app		
01 – 10	96.4% (n=270)	96.6% (n=397)
11 – 20	1.8% (n=5)	2.9% (n=12)
21 – 30	0.7% (n=3)	0.5% (n=2)
> 30	1.1% (n=3)	-
Target population		
<18 years	96.4% (n=270)	95.6% (n=393)
>18 years	3.6% (n=10)	4.1% (n=17)
No information	-	0.2% (n=1)
Investment for use		
Free	25.7% (n=72)	12.2% (n=50)
Payment	52.1% (n=146)	65.5% (n=269)
No information	22.1% (n=62)	22.4% (n=92)
Prices		
Up to US$11.99	45.2% (n=66)	43.5% (n=117)
US$12 - $23,99	6.8% (n=10)	11.5% (n=31)
>US$24	6.8% (n=10)	12.6% (n=34)
No information	41.1% (n=60)	32.3% (n=87)
Seller		
One app	94% (n=236)	90.1% (n=317)
Two apps	3.6% (n=9)	8% (n=28)

Three apps	0.8% (n=2)							0.9% (n=3)
Four apps	0.8% (n=2)							0.3% (n=1)
> four apps	0.8% (n=2)							0.9% (n=3)
***Number of reviews* [a]**	**iOS device**		**Apple (Website)**		**Android device**		**Play Store (Website)**	
< 9.999	40% (n=111)	36% (n=148)	40% (n=112)	33.8% (n=139)	17.1% (n=48)	20.2% (n=83)	35.4% (n=99)	29.9% (n=123)
10.000 – 19.999	1% (n=3)	1.2% (n=5)	1% (n=4)	2.2% (n=9)	3.2% (n=9)	1.5% (n=6)	2.9% (n=8)	1.5% (n=6)
> 20.000	3% (n=8)	6.3% (n=26)	3.2 (n=9)	7.5% (n=31)	4.3% (n=12)	8% (n=33)	7.1% (n=20)	10.5% (n=43)
No information	56% (n=158)	56.4% (n=232)	55.4% (n=155)	56.4% (n=232	75.4% (n=211)	70.3% (n=289)	54.6% (n=153)	58.2% (n=239)
***Number of downloads* [b]**								
Up to 9.999	22.9% (n=64)							23.6% (n=97)
10.000 – 19.999	11.1% (n=31)							14.4% (n=59)
> 20.000	41.4% (n=116)							42.6% (n=175)
No information	24.6% (n=69)							19.5% (n=80)
***Consumer rating* [c]**	**iOS device**		**Apple (Website)**		**Android device**		**Play Store (Website)**	
≤3.0	16.1% (n=45)	5.4% (n=22)	2.9% (n=8)	0.7% (n=3)	2.9% (n=8)	3.4% (n=14)	2.1% (n=6)	1.7% (n=7)
3.1 – 3.9	5% (n=14)	1.7% (n=7)	0.4% (n=1)	0.2% (n=1)	3.6% (n=10)	3.2% (n=13)	6.1% (n=17)	4.9% (n=20)
>4.0	50% (n=140)	44.3% (n=182)	42.5% (119)	44.5% (n=183)	18.2% (n=51)	22.9% (n=94)	27.1% (n=76)	31.6% (n=130)
No information	28.9% (n=81)	48.7% (n=200)	54.3% (n=152)	54.5% (n=224)	75.4% (n=211)	70.6% (n=290)	64.6% (n=181)	61.8% (n=254)
Technical support								
Yes	96.8% (n=271)							94.4% (n=388)
No	2.9% (n=8)							5.1% (n=21)
No information	0.4% (n=1)							0.5% (n=2)

[a, b, c] Due to divergent values, four values were inserted. The information was collected on mobile devices with iOS and Android systems and on the official websites of the Google Store and Apple Store. Data are presented in the following order: iOS device, Apple (Website), Android device, and Play Store (Website).
